# Does Mucosal B1 Activation Result in the Accumulation of Peak IgM During Chronic Intrarectal SIVmac239 Exposure to Protect Chinese-Origin Rhesus Macaques From Disease Progression?

**DOI:** 10.3389/fmicb.2020.00357

**Published:** 2020-03-19

**Authors:** Zhe Cong, Ling Tong, Yuhong Wang, Aihua Su, Ting Chen, Qiang Wei, Jing Xue, Chuan Qin

**Affiliations:** ^1^Beijing Key Laboratory for Animal Models of Emerging and Remerging Infectious Diseases, NHC Key Laboratory of Human Disease Comparative Medicine, Institute of Laboratory Animal Science, Chinese Academy of Medical Sciences and Comparative Medicine Center, Peking Union Medical College, Beijing, China; ^2^Department of Gerontology and Geriatrics, The First Affiliated Hospital of Harbin Medical University, Harbin, China

**Keywords:** PBMC, LPMC, SIV, IgM, B1 cells, Chinese-origin rhesus macaques, disease progression

## Abstract

Human immunodeficiency virus (HIV) infection is characterized by a dynamic process and highly variable progression. Although extensive comparisons have been reported between the minority of non-progressors (NPGs) and the majority of progressors (PGs), the underlying mechanism is still unclear. One reason for this is that the initial onset of infection is very difficult to track, particularly when men who have sex with men (MSM) are predominantly responsible for the transmission of human HIV. To find potential early protection strategies against later progression during chronic mucosal exposure, 10 Chinese-origin rhesus macaques (ChRhs) that underwent repetitive simian immunodeficiency virus (SIV) intrarectal exposure were longitudinally tracked. The results of the periodic detection of peripheral blood mononuclear cells (PBMCs) and colorectal mucosal lamina propria mononuclear cells (LPMCs) with immunoglobulins in rectal fluid were compared between non-progressive and progressive subgroups, which were classified based on their circulating viral loads. As a result, four NPGs and six PGs were observed after disease onset for 2 months. Upon comparing the mucosal and systemic immune responses, the PBMC response did not differ between the two subgroups. Regarding LPMCs, the increased activation of B1a/B1 cells among B cells and a peak in IgM in rectal fluid was observed approximately 10 days after the first exposure, followed by consistently low viremia in the four non-progressive ChRhs. In the six progressive ChRhs, neither B cell activation nor a peak in IgM was observed, while a robust elevation in IgG was observed, followed by consistently high viremia post exposure. Based on the PBMC-LPMC disparity between the subgroups of monkeys, we hypothesize that early B1 activation in LPMCs that result in an IgM peak might attenuate the entry and acquisition of SIV in the mucosa, resulting in very low dissemination into blood. Our models have suggested that the use of early surveillance both systemically and in the mucosa to comprehensively determine virus–host interactions would be informative for mucosal vaccine development.

## Introduction

A tiny proportion of humans exposed to human immunodeficiency virus (HIV) retain a long-term non-progressive status and are known as “long-term non-progressors” (LTNPs) or “elite controllers” (ECs). Compared to the majority of those infected with HIV, who produce a strong virus-specific immune response and show obvious disease characteristics, LTNPs demonstrate extraordinarily low viral loads and do not progress to illness despite a lack of antiretroviral treatment (Sahu et al., 2001, 2005). More than 90% of new HIV infections occur by sexual transmission through mucosal contact (Tebit et al., 2012). Men who have sex with men (MSM) have the highest risk of HIV transmission and continue to transmit new HIV infections worldwide. Repetitive mucosal exposures in MSM could substantially enhance the frequency of mucosal immunity (Hladik and McElrath, 2008). Therefore, spontaneous LTNPs or ECs who are also MSM could provide a natural model of HIV-host immunity interactions. However, despite intensive studies comparing “dichotomized” infectors (Bendenoun et al., 2018), the mechanisms of “natural” protective mechanisms have not been clarified (Gurdasani et al., 2014). When considering the scenario, mucosal immunity as the first barrier (Miller et al., 1989; Xu et al., 2013) is the primary step in HIV infection, even in cases where infection occurs by the intravenous route (Bolton et al., 2012; Sutton et al., 2016). Therefore, early mucosal immunity, which could potentially confer some protection to non-progressed MSM infectors, has not been revealed because it is extremely difficult to detect the primary response to mucosal exposure during HIV transmission. In the present study, we employed Chinese-origin rhesus macaques (ChRhs) to generate spontaneous viral controllers with repeated low-dose simian immunodeficiency virus (SIV) exposure by the mucosal route. The animals were observed and the number of SIV RNA copies was detected periodically. NHP monkeys were divided into non-progressors (NPGs) and progressors (PGs) based on the degree of viral replication. By tracking primary mucosal immunity, immune responses occurring from the entry point in the rectal mucosa to the peripheral blood were compared in parallel to find some informative clues about how the first barrier of mucosal immunity performs in future non-progressive controllers.

## Materials and Methods

### Animals and Ethics Statement

Two- to 4-year-old male and female ChRhs (*Macaca mulatta*) were housed and cared for in accordance with the Care and Use of Laboratory Animals of the Institute of Laboratory Animal Science and the recommendations of the welfare report for the use of non-human primates in research^1^ in an Association for the Assessment and Accreditation of Laboratory Animal Care (AAALAC)-accredited facility. All macaques used in this study tested negative for the major histocompatibility complex class I (MHC-I) Mamu-A^∗^01, Mamu-A^∗^02, Mamu-B^∗^08, and Mamu-B^∗^17 alleles (Silver and Watkins, 2017) to reduce the bias introduced by MHC-enhanced control on SIV replication (Silver and Watkins, 2017). All animal procedures and experiments were performed according to protocols approved by the Institutional Animal Care and Use Committee (IACUC) of the Institute of Laboratory Animal Science, Chinese Academy of Medical Sciences (No. ILAS-VL-2012-002 and No. ILAS-VL-2015-003). All animals were anesthetized with an intramuscular injection of 10 mg/kg ketamine hydrochloride prior to sample collection, and the experiments were performed in the biosafety level 3 laboratory.

### Animal Model and Longitudinal Tracing

**FIGURE 1:**
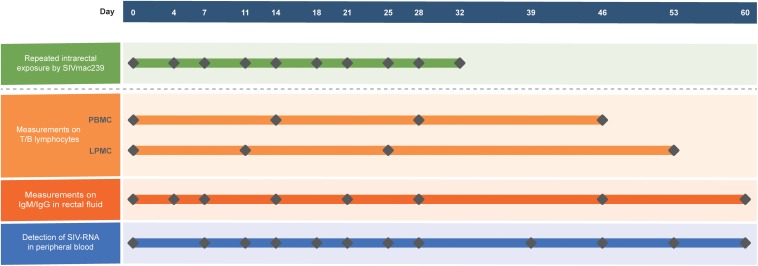
Generation of the model using 10 ChRhs with periodical detection in the circulation and gut. Ten ChRhs were intrarectally exposed to low doses of SIVmac239 twice a week 10 times (along the green bar). T and B lymphocytes were measured at four time points in PBMCs or LPMCs at a short interval to minimize the impairment of the animals (along the light orange bar). Eight measurements of IgM and IgG were carried out in rectal fluid (along the dark orange bar). The plasma viral loads were detected 10 times (along the blue bar).

Ten ChRhs were exposed intrarectally to SIVmac239 at a median tissue culture infectious dose (TCID_50_) of 100 twice a week for 5 weeks. The SIVmac239 strain used was kindly gifted by Dr. Preston Marx at the Aaron Diamond AIDS Research Center of the United States. For the longitudinal tracking of virus-host immunity, peripheral blood, intestinal biopsy specimens, and rectal fluid were collected prior to and after the designated virus exposure. The animal model and longitudinal sampling schedule are shown in Figure 1. During the 60-day observation period, peripheral blood mononuclear cells (PBMCs) were collected at four time points. Colorectal mucosal lamina propria mononuclear cells (LPMCs) were biopsied by using an endoscope four times before or after PBMCs collection for the sake of animal welfare (McGary et al., 2017). Rectal fluid was collected at eight time points. The viral load in peripheral blood was intensively detected at twelve time points in each exposed monkey.

### qRT-PCR Assay

Plasma RNA was extracted and purified using a QIAamp Viral RNA Mini Kit (Qiagen, Valencia, CA, United States). The quantification of plasma viral RNA in each sample was performed by quantitative real-time reverse transcription-PCR (qRT-PCR) using primers specific to a conserved region in the SIVmac239 genome. The primers and probes used for vRNA amplification were gag91 forward (GCAGAGGAGGAAATTACCCAGTAC), gag91 reverse (CAATTTTACCCAGGCATTTAATGTT), and pSIVgag91-1 (5′-FAM-ACCTGCCATTAAGCCCGA-MGB-3′) (Chong et al., 2018). The limit of detection was 100 copy equivalents of RNA per ml of plasma. Three test reactions were performed for each sample.

### Flow Cytometry to Measure T/B Cell Shifting in PBMCs/LPMCs

Gut biopsies were performed as previously described (Xue et al., 2013), and the biopsy tissue was then treated with 5 mM EDTA and 60 U/ml collagenase. LPMCs were enriched for lymphocytes by Percoll density gradient centrifugation, and PBMCs were isolated using conventional Ficoll Hypaque density gradient centrifugation (GE Healthcare, Uppsala, Sweden). Polychromatic flow cytometry was performed to stain the T lymphocyte panel or B lymphocyte panel. For the T lymphocyte panel, 50 μl of EDTA-anticoagulated whole blood or 1 × 10^6^ primary cells derived from the lamina propria were stained with the monoclonal antibodies CD3-PE/Cy7 (SP34-2), CD4-Percp/Cy5.5 (L200), and CD8-APC/Cy7 (RPA-T8) from BD Biosciences (San Jose, CA, United States). The CD4^+^ T cell counts were determined with BD Truecount tubes according to the manufacturer’s instructions (BD, San Jose, CA, United States). For the B lymphocyte panel, PBMCs or LPMCs were incubated with CD3-PE/Cy7 (SP34-2) from BD Biosciences (San Jose, CA, United States); CD20-FITC (2H7), CD43-APC (10G7), and CD27-APC/Cy7 (O323) from Biolegend (San Diego, CA, United States); and CD5- PerCP/Cy5.5 (5D7) from Invitrogen (Carlsbad, CA, United States) to determine the following B cell subsets (Brocca-Cofano et al., 2017): B cells, CD3^–^CD20^+^; B1 cells, CD3^–^CD20^+^CD43^+^CD27^+^; and B1a cells, CD3^–^CD20^+^CD43^+^CD27^+^CD5^+^. All the samples were analyzed by flow cytometry (FACSAria; BD, CA, United States).

### Purification of IgM and IgG in Rectal Fluid

IgM in rectal fluid was purified with HiTrap^TM^ IgM Purification HP according to the manufacturer’s instructions (GE, Boston, MA, United States) (Hara et al., 2013). IgG in rectal fluid was purified with Protein A agarose resin. Briefly, the Protein A agarose resin was washed and equilibrated with PBS at pH 7.5. Then, filtered rectal fluid from each monkey was loaded onto the column at a flow rate of 0.2 ml/min. The adsorbed materials were eluted with 0.1 M glycine buffer at pH 2.5 after washing the column with PBS at a flow rate of 1 ml/min. The eluted fractions were neutralized with phosphate buffer at pH 8.5 and the effluents were monitored by UV spectrometry at 280 nm.

### Statistical Analysis

Mann–Whitney *U*-tests were used to compare the plasma viral loads of non-progressive and progressive monkeys. Comparisons between two subgroups were determined using the unpaired *t*-test (Welch). Correlations between two variables were assessed by the Spearman correlation. All statistical analyses were performed with GraphPad Prism 6.0 software (GraphPad Software Inc., San Diego, CA, United States).

## Results

### Generation of a Chronic Progression-Dichotomized Model Using 10 Homogenous ChRhs

**FIGURE 2:**
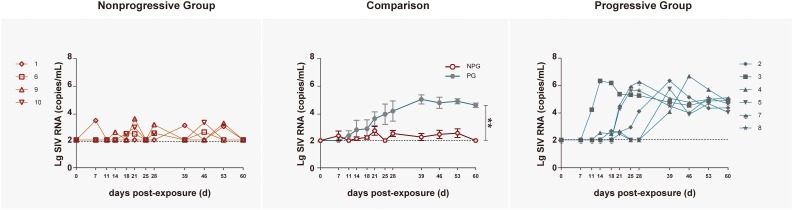
Tracking plasma viral loads by detecting SIV RNA in non-progressive and progressive monkeys. The log of the geometric mean of the number of plasma SIVmac239 copies was compared between four non-progressive monkeys (orange line) and six progressive monkeys (blue line). The plasma viral loads are shown as log10 copies/ml. The assay had a sensitivity of 100 viral RNA copies per ml of plasma (black dotted lines). According to the Mann–Whitney *U*-test, the viral load in progressive monkeys was significantly increased compared with that in non-progressive monkeys (**P < 0.01).

To track the immune responses of monkey models between NPGs and PGs in parallel, 10 ChRhs were inoculated intrarectally with SIV (McDermott et al., 2004). The age, weight, white blood cell count in peripheral blood, and daily function score (Deng et al., 2019) were comparable to those at baseline (data not shown). The methods of repetitive mucosal exposure and sampling of peripheral blood, gut tissue, and rectal fluid were carried out on schedule (Figure 1). According to viral copy detection in peripheral blood, six ChRhs (Nos. 2, 3, 4, 5, 7, and 8) developed into chronic SIV PGs with stable high viremia and mild variation. By using peak plasma viremia to determine the degree of viral replication, the average number of copies was determined to be approximately 10^6^ copies/ml with a range from 5.55 to 6.59 log_10_ RNA copies/ml. The remaining ChRhs (Nos. 1, 6, 9, and 10) were found to be NPGs, as the peak plasma viremia of SIVmac239 in these monkeys was consistently lower than 10^4^ copies/ml throughout the entire observation period (Figure 2). During the follow-up observation period of 1 year, viremia in the four non-progressive monkeys remained as low as approximately 2–4 log_10_ RNA copies/ml (data not shown). Therefore, the non-progressive monkeys successfully mimicked the “long-term HIV controllers,” and the progressive monkeys mimicked the chronic HIV infectors.

### T/B Lymphocyte Activation in PBMCs and LPMCs in Non-Progressive and Progressive Monkeys

**FIGURE 3:**
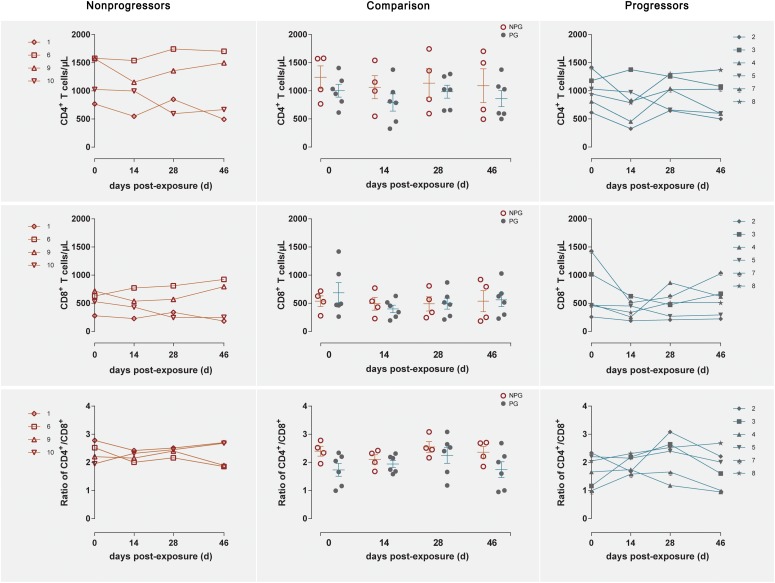
Changes in T lymphocytes among PBMCs between non-progressive and progressive monkeys. The CD4^+^ T cell counts, CD8^+^ T cell counts, and CD4^+^/CD8^+^ ratios were measured in peripheral blood at four time points in four non-progressive monkeys and six progressive monkeys. No significant differences were found between the two subgroups at any time point, indicating that no notable T cell changes occurred in PBMCs from ChRhs subjected to repetitive SIV mucosal exposure (unpaired *t*-test, Welch’s correction, *P* > 0.05).

**FIGURE 4:**
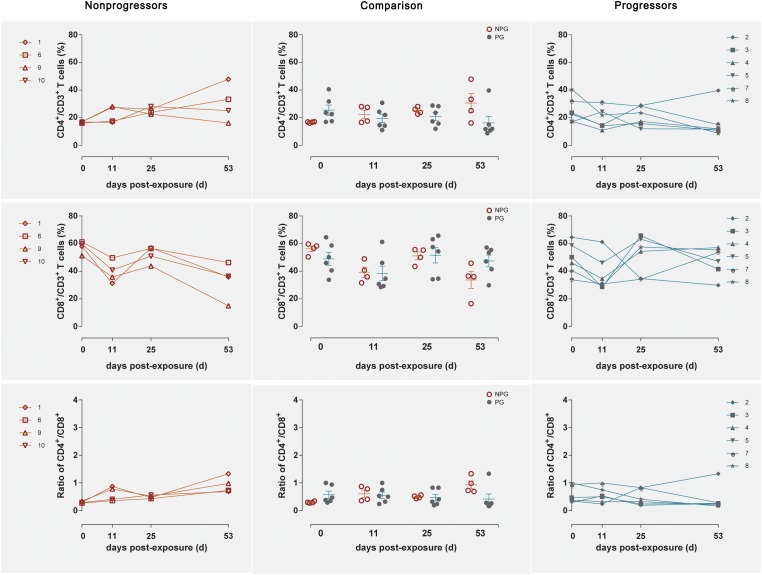
Changes in T lymphocytes among LPMCs in non-progressive and progressive monkeys. The CD4^+^ T cell percentages, CD8^+^ T cell percentages, and CD4^+^/CD8^+^ ratios were measured in LPMCs obtained at four time points in four non-progressive monkeys and six progressive monkeys. No significant differences were found between the two subgroups at any time point, indicating that no notable T cell changes occurred in LPMCs from ten ChRhs subjected to repetitive SIV mucosal exposure (unpaired *t*-test, Welch’s correction, *P* > 0.05).

Longitudinal changes in the CD4^+^ T cell counts, CD8^+^ T cell counts, and CD4^+^/CD8^+^ T cell ratios in peripheral blood were demonstrated in the four non-progressive monkeys and six progressive monkeys (Figure 3). Comparisons between the two subgroups revealed no changes in the CD4^+^ T cell counts, CD8^+^ T cell counts, or CD4^+^/CD8^+^ T cell ratios at any of the four detection times between NPGs and PGs (*P* ¿ 0.05). A notably higher average CD4^+^/CD8^+^ T cell ratio was observed in NPGs at baseline, although statistical significance was not maintained (*P* = 0.068), indicating that measurement of the baseline T lymphocytes in peripheral blood could not be used to predict the outcome of the shift in T lymphocyte activation. For LPMCs, four monkeys among the NPGs and six monkeys among the PGs also showed a similar tendency in terms of the T lymphocyte shift after repeated low-dose SIV challenge (Figure 4). The percentages of CD4^+^ T cells, percentages of CD8^+^ T cells, and CD4^+^/CD8^+^ T cell ratios were comparable between NPGs and PGs at each observation point (*P* ¿ 0.05).

**FIGURE 5:**
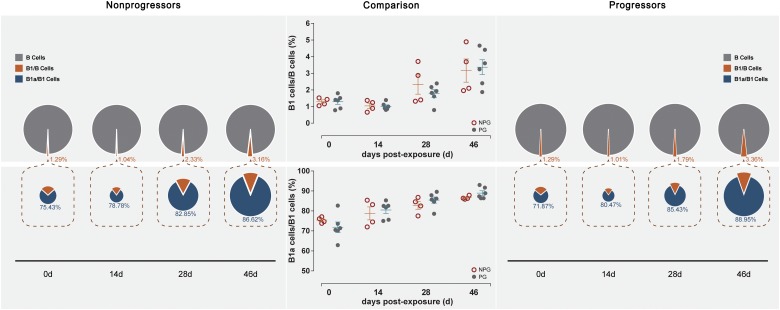
Changes in B lymphocytes in PBMCs between non-progressive and progressive monkeys. B cell subsets, including B1/B and B1a/B1, were measured from PBMCs at four time points in four non-progressive monkeys and six progressive monkeys. The percentages of B1/B and B1a/B1 cells were compared between the two subgroups at each time point. No significant changes in B cells occurred in PBMCs in ten ChRhs under repetitive SIV mucosal exposure (unpaired *t*-test, Welch’s correction, *P* > 0.05).

**FIGURE 6:**
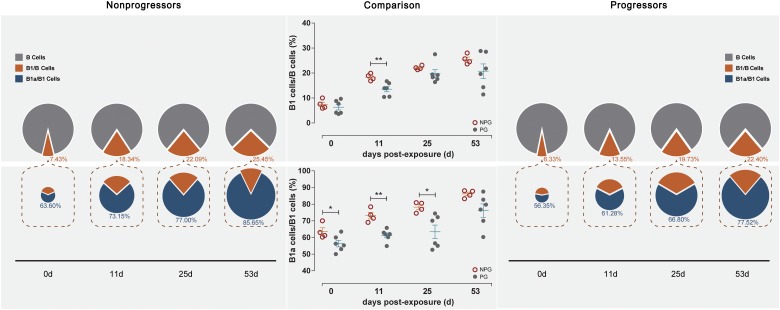
Changes in B lymphocytes among LPMCs in non-progressive and progressive monkeys. B cell subsets, including B1/B and B1a/B1 cells, were measured among LPMCs obtained at four time points in four non-progressive monkeys and six progressive monkeys. The percentages of B1/B and B1a/B1 cells were compared between the two subgroups at each time point. Notably, increased ratios of B1/B and B1a/B1 cells were found in the four non-progressive monkeys during the early stage after initial exposure (unpaired *t*-test, Welch’s correction, *P < 0.05, **P < 0.01).

Next, the activation of B lymphocytes was examined between the two subgroups. In PBMCs, the B cell subset shift remained stable, including that of B1 cells among B cells and B1a cells among B1 cells, between NPGs and PGs. In addition, compared to the shifting of T cell subsets, the longitudinal shifting of B cell subsets was more stable, indicating that B lymphocyte activation in PBMCs was minimally impacted during SIV mucosal exposure in ChRhs (Figure 5). However, in LPMCs, the percentage of B1a cells among B1 cells and that of B1 cells among B cells from mucosa was significantly increased in non-progressive monkeys compared with progressive monkeys at the first detection. Additionally, dramatically increased B1/B and B1a/B1 cell ratios were observed in the early stage (approximately 11 days after the initial challenge) in the NPGs compared to the PGs (Figure 6). The increased numbers of B1a/B1 cells in LPMCs from NPGs were observed for 25 days after the initial exposure. At 53 days post exposure, a non-significant difference in the B cell shift was shown between the two progression-distinct subgroups. Longitudinally, the peaks in the increased B1 and B1a cells indicated the transient activation of B lymphocytes in LPMCs during the early stage in the four non-progressive monkeys.

### Expression of IgM and IgG in Rectal Fluid From 10 Monkeys

**FIGURE 7:**
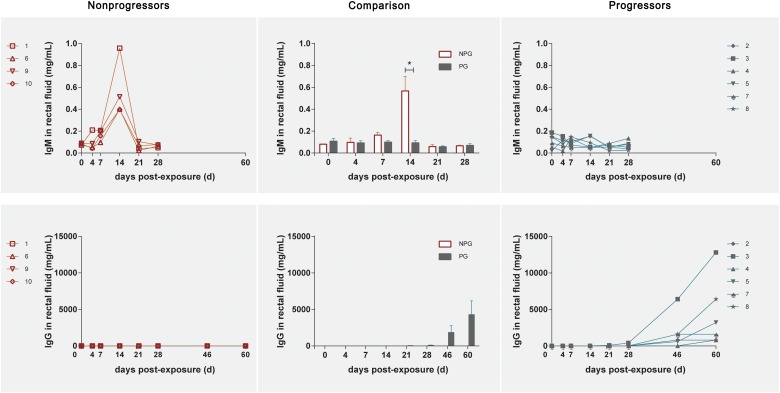
Levels of IgM and IgG in rectal fluid in non-progressive and progressive monkeys. The levels of IgM and IgG were detected after initial exposure. A precipitously elevated IgM level in rectal fluid was found in four non-progressive monkeys during the early stage after initial exposure. In six progressive monkeys, a gradual increase in IgG was observed during the late stage 2 weeks after the first exposure. According to unpaired t test results, the level of IgM in the four non-progressive monkeys was significantly higher than that in the six progressive monkeys, whereas the level of IgG in the six progressive monkeys was significantly higher than that in the four non-progressive monkeys (*P < 0.01).

In NPGs, IgM in rectal fluid precipitously increased to an average of 0.56 mg/ml with a range of 0.397–0.958 mg/ml at 14 days postexposure and gradually decreased to baseline levels at 21 days postexposure. Undetectably low IgM levels were observed during the entire observation period in the six PGs. An elevated IgG level in rectal fluid was shown at 46 days postexposure, and this level plateaued until 60 days (Figure 7).

**FIGURE 8:**
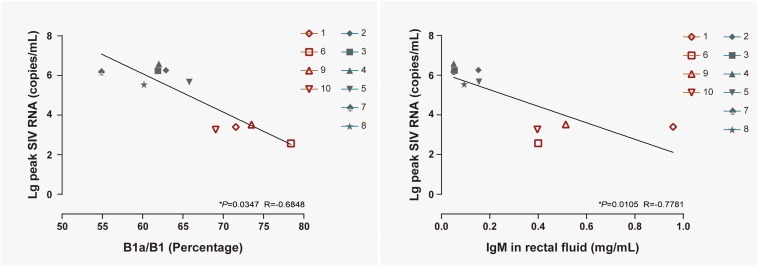
Correlation of the B1a/B1 cell ratio among LPMCs and the IgM level in rectal fluid with peak viremia in 10 ChRhs. The correlations of peak viremia with the B1a/B1 cell ratio among LPMCs detected on day 11 post infection (left panel) and the level of IgM in rectal fluid detected at day 14 post infection (right panel) are shown. Using the Spearman correlation, strong significant linear correlations were observed between the B1a/B1 cell ratio and peak viremia (*R* = −0.6848) and between the level of IgM in rectal fluid and peak viremia (*R* = −0.7781) in 10 ChRhs with mucosal SIV exposure (*P < 0.05).

B1 cells have been identified as a major source of infection-induced local IgM (Choi and Baumgarth, 2008; Baumgarth, 2011). To reveal the correlation between B cell activation in LPMCs, at 14 days postexposure the level of IgM in rectal fluid, viral replication in peripheral blood, B1a/B1 ratio, and IgM levels were individually correlated with the peak viral load in ten monkeys, including both NPGs and PGs. A temporally significant correlation was observed between the B1a/B1 ratio in LPMCs (*P* < 0.05, *R* = −0.6848) or the level of IgM in rectal fluid (*P* < 0.05, *R* = −0.7781) at 14 days postexposure and the peak viral load during the observation (Figure 8). As B1 cells contribute substantially to mucosal defense against pathogens, the presence of both negative correlations indicated that stepwise activation of B1 cells with subsequent peak IgM production in LPMCs might be responsible for the suppressed viral load in non-progressive monkeys. Therefore, we hypothesized that transient mucosal B1 cell activation along with peak IgM production in LPMCs impeded viral progression in SIV-infected monkeys.

## Discussion

We tracked the stepwise progression/non-progression of chronic mucosal exposure to SIV in ChRhs, which could mimic the most prevalent route of HIV transmission. It has been documented that ChRhs are relatively resistant to SIV-related progression, with approximately 30% of infected monkeys maintaining very low viral loads for several years (Ling et al., 2002). Therefore, the progression-dichotomized statuses of these homogenous monkeys could be easily predicted. In the present study, monkeys with genes highly associated with SIV spontaneous controllers, such as the MHC-I alleles Mamu-A^∗^01 (Pal et al., 2002; Lim et al., 2010), Mamu-A^∗^02 (Loffredo et al., 2004), Mamu-B^∗^08 (Loffredo et al., 2007), and Mamu-B^∗^17 (Yant et al., 2006), were removed from the study. Therefore, the MHC-dependent disparity has been minimized. As no well-acknowledged criteria for the viral load defining ECs/LTNPs/PGs status have been established in the ChRh model, as they have in HIV-infected humans, we simply classified the monkeys as either NPGs or PGs in the ChRh model. The dose used in this study could be considered low according to our experience. Indeed, even a TCID_50_ of 50,000 could produce non-progressive controllers. As low- and high-dose challenges produced a similar PG/controller ratio (McDermott et al., 2004; Tasca et al., 2007; Xiao et al., 2012; Greene et al., 2014), the initial acquisition very likely played a predominant role in later outcomes. Based on the longitudinal viral load trajectories, four monkeys were observed to be NPGs, while six monkeys were classified as PGs among the 10 ChRhs. Therefore, we could use the present SIV ChRh model to mimic the general immunity disparity between the minority of NPGs/ECs and the majority of PGs in humans with HIV. Another characteristic of our model was that it could be used to track the very early stage of chronic mucosal infection with parallel detection in LPMCs and PBMCs. In humans, it is very difficult to track the onset of a chronic infection by observing both systemic and mucosal immunity (Lama et al., 2016). Here, we intensively tracked both PBMCs and LPMCs for T and B cell activation as well as IgM and IgG expression from rectal fluid, as it has been shown that the levels of immunoglobins in rectal fluid could robustly reflect the concentrations in mucosal tissues (Cottrell et al., 2016). A notable limitation was that IgA could not be detected despite numerous attempts to purify IgA from monkeys.

Our tracking observation resulted in an interesting difference between NPGs and PGs in the early stage post exposure. B1 cell activation (Choi and Baumgarth, 2008; Baumgarth, 2011), along with a precipitous increase in IgM, was observed in LPMCs at only approximately 9–11 days after the initial exposure in the four non-progressive monkeys. Our results are consistent with the phenomenon that early treatment with anti-HIV neutralizing antibodies potently induces sustainable immunity and subsequent progression (Nishimura et al., 2017). Although neutralizing antibodies showed a superior ability to block viral infection compared to that of non-neutralizing antibodies (Cheeseman et al., 2017), a paradoxical phenomenon showed that non-neutralizing antibodies were much more commonly detected in HIV ECs than neutralizing antibodies (Theze et al., 2011). In addition, a mucosal vaccine was found to have protective efficacy independent of anti-HIV neutralizing antibodies (Sui et al., 2019). Therefore, determination of the extent to which the early IgM peak contributes to the substantial suppression of viral dissemination within the mucosa in NPGs requires further experiments. As a non-specific antibody, natural IgM has been shown to robustly neutralize viruses within the mucosal epithelial barrier *in vitro* (Devito et al., 2018). After a peak in IgM was induced in LPMCs, viremia remained consistently low in the four NPGs, and the timing was consistent with that of the viremia peak during infection via the intrarectal route (Haseltine, 1989). Over 14 days, a stepwise route of infection from the mucosal epithelial barrier to the peripheral blood via profound mucosal immunity was observed (Miller et al., 2005; Haase, 2010). In this scenario, we supposed that the peak in IgM that occurred after approximately 10 days might have been an accumulative response to repetitive SIV exposure. Within the mucosa, gut-associated lymphoid tissue (GALT) made up of organized lymphoid nodules and multifunctional LPMCs could mount profound immunity responses both cellularly and humorally. Therefore, the LPMC response showed a sufficiently high efficacy to produce an EC without this response being mirrored in the blood (Shacklett and Ferre, 2011). Moreover, colorectal tissue was proven to be a consistent viral reservoir in long-term non-progressive ChRhs (Ling et al., 2010). Therefore, the disparity between the responses of NPGs and PGs has strongly demonstrated the fact that “outcomes depend on a race between expansion of infection and the immune response generated to contain it” (Li et al., 2009) in the early stages of viral infection.

Taken together, our results support the hypothesis that an accumulative mucosal immune response induced by repetitive antigenic stimulation results in a peak IgM response in LPMCs. This mechanism may be the reason that ∼30% of ChRhs were protected from dissemination and led to their becoming NPGs. The exposure-dependent immune response was mild yet accumulative and produced a robust non-specific IgM to neutralize insufficient antigens, followed by the blockage of dissemination. We hypothesize that early B1 cell activation along with the IgM peak in LPMCs might exert a “decapacitating effect” to protect ChRhs from progression. The primary cause of this result was the characterized genotypes, which were characterized as central to the PG. On the other hand, susceptible animals who acquired SIV infection, albeit at a low dose, gradually developed an antigen-specific IgG response, and subsequent viral dissemination could not be avoided. The heterogeneity between individuals whose minority could be initiated with minimal acquisition of the virus might be the determining cause of the remaining NPGs. Additional studies will be needed to explore the effect of the interaction between individual genotypes on the GALT-IgM response in ChRhs to determine the underlying mechanism.

## Data Availability Statement

All datasets generated for this study are included in the article/supplementary material.

## Ethics Statement

The animal study was reviewed and approved by the Institutional Animal Care and Use Committee (IACUC) at the Institute of Laboratory Animal Science, Chinese Academy of Medical Sciences (No. ILAS-VL-2012-002 and No. ILAS-VL-2015-003).

## Author Contributions

JX and CQ contributed to conceptualization, resources, and supervision. ZC and LT contributed to methodology. ZC, LT, AS, and TC contributed to investigation. LT, YW, and JX contributed to writing the original draft. YW and JX contributed to writing—review and editing. ZC, JX, and QW contributed to funding acquisition.

## Conflict of Interest

The authors declare that the research was conducted in the absence of any commercial or financial relationships that could be construed as a potential conflict of interest.

## Footnotes

1 http://www.acmedsci.ac.uk/more/news/the-use-of-non-human-primates-in-research/

## References

[B1] Baumgarth N. (2011). The double life of a B-1 cell: self-reactivity selects for protective effector functions. *Nat. Rev. Immunol.* 11 34–46. 10.1038/nri2901 21151033

[B2] Bendenoun M. Samri A. Avettand-Fenoel V. Cardinaud S. Descours B. Carcelain G. (2018). What is the most important for elite control: genetic background of patient, genetic background of partner, both or neither? Description of complete natural history within a couple of MSM. *EBioMedicine* 27 51–60. 10.1016/j.ebiom.2017.12.003 29273355 PMC5828297

[B3] Bolton D. L. Song K. Wilson R. L. Kozlowski P. A. Tomaras G. D. Keele B. F. (2012). Comparison of systemic and mucosal vaccination: impact on intravenous and rectal SIV challenge. *Mucosal Immunol.* 5 41–52. 10.1038/mi.2011.45 22031182 PMC3732474

[B4] Brocca-Cofano E. Kuhrt D. Siewe B. Xu C. Haret-Richter G. S. Craigo J. (2017). Pathogenic correlates of simian immunodeficiency virus-associated B cell dysfunction. *J. Virol.* 91:e1051-17. 10.1128/JVI.01051-17 28931679 PMC5686751

[B5] Cheeseman H. M. Olejniczak N. J. Rogers P. M. Evans A. B. King D. F. L. Ziprin P. (2017). Broadly neutralizing antibodies display potential for prevention of HIV-1 infection of mucosal tissue superior to that of nonneutralizing antibodies. *J. Virol.* 91:e1762-16. 10.1128/JVI.01762-16 27795431 PMC5165208

[B6] Choi Y. S. Baumgarth N. (2008). Dual role for B-1a cells in immunity to influenza virus infection. *J. Exp. Med.* 205 3053–3064. 10.1084/jem.20080979 19075288 PMC2605232

[B7] Chong H. Xue J. Zhu Y. Cong Z. Chen T. Guo Y. (2018). Design of novel HIV-1/2 fusion inhibitors with high therapeutic efficacy in rhesus monkey models. *J. Virol.* 92:e775-18. 10.1128/JVI.00775-18 29899103 PMC6069194

[B8] Cottrell M. L. Prince H. M. Allmon A. Mollan K. R. Hudgens M. G. Sykes C. (2016). Cervicovaginal and rectal fluid as a surrogate marker of antiretroviral tissue concentration: implications for clinical trial design. *J. Acquir. Immune Defic. Syndr.* 72 498–506. 10.1097/QAI.0000000000000996 26999532 PMC4942408

[B9] Deng W. Guan G. Xiao C. Qu G. Xue J. Qin C. (2019). Construction of a comprehensive observer-based scale assessing aging-related health and functioning in captive rhesus macaques. *Aging (Albany NY)* 11 6892–6903. 10.18632/aging.102219 31498777 PMC6756902

[B10] Devito C. Ellegard R. Falkeborn T. Svensson L. Ohlin M. Larsson M. (2018). Human IgM monoclonal antibodies block HIV-transmission to immune cells in cervico-vaginal tissues and across polarized epithelial cells in vitro. *Sci. Rep.* 8:10180. 10.1038/s41598-018-28242-y 29977063 PMC6033918

[B11] Greene J. M. Weiler A. M. Reynolds M. R. Cain B. T. Pham N. H. Ericsen A. J. (2014). Rapid, repeated, low-dose challenges with SIVmac239 infect animals in a condensed challenge window. *Retrovirology* 11:66. 10.1186/s12977-014-0066-z 25125288 PMC4149191

[B12] Gurdasani D. Iles L. Dillon D. G. Young E. H. Olson A. D. Naranbhai V. (2014). A systematic review of definitions of extreme phenotypes of HIV control and progression. *AIDS* 28 149–162. 10.1097/QAD.0000000000000049 24149086 PMC3882304

[B13] Haase A. T. (2010). Targeting early infection to prevent HIV-1 mucosal transmission. *Nature* 464 217–223. 10.1038/nature08757 20220840

[B14] Hara A. Furuichi K. Higuchi M. Iwata Y. Sakai N. Kaneko S. (2013). Autoantibodies to erythropoietin receptor in patients with immune-mediated diseases: relationship to anaemia with erythroid hypoplasia. *Br. J. Haematol.* 160 244–250. 10.1111/bjh.12105 23151030

[B15] Haseltine W. A. (1989). Silent HIV infections. *N. Engl. J. Med.* 320 1487–1489. 10.1056/nejm198906013202210 2716799

[B16] Hladik F. McElrath M. J. (2008). Setting the stage: host invasion by HIV. *Nat. Rev. Immunol.* 8 447–457. 10.1038/nri2302 18469831 PMC2587276

[B17] Lama J. R. Karuna S. T. Grant S. P. Swann E. M. Ganoza C. Segura P. (2016). Transient peripheral immune activation follows elective sigmoidoscopy or circumcision in a cohort study of MSM at risk of HIV infection. *PLoS One* 11:e0160487. 10.1371/journal.pone.0160487 27536938 PMC4990246

[B18] Li Q. Skinner P. J. Ha S. J. Duan L. Mattila T. L. Hage A. (2009). Visualizing antigen-specific and infected cells in situ predicts outcomes in early viral infection. *Science* 323 1726–1729. 10.1126/science.1168676 19325114 PMC2753492

[B19] Lim S. Y. Chan T. Gelman R. S. Whitney J. B. O’Brien K. L. Barouch D. H. (2010). Contributions of Mamu-A^∗^01 status and TRIM5 allele expression, but not CCL3L copy number variation, to the control of SIVmac251 replication in Indian-origin rhesus monkeys. *PLoS Genet.* 6:e1000997. 10.1371/journal.pgen.1000997 20585621 PMC2891712

[B20] Ling B. Mohan M. Lackner A. A. Green L. C. Marx P. A. Doyle L. A. (2010). The large intestine as a major reservoir for simian immunodeficiency virus in macaques with long-term, nonprogressing non-progressing infection. *J. Infect. Dis.* 202 1846–1854. 10.1086/657413 21050120 PMC3058301

[B21] Ling B. Veazey R. S. Luckay A. Penedo C. Xu K. Lifson J. D. (2002). SIV(mac) pathogenesis in rhesus macaques of Chinese and Indian origin compared with primary HIV infections in humans. *AIDS* 16 1489–1496. 10.1097/00002030-200207260-00005 12131186

[B22] Loffredo J. T. Maxwell J. Qi Y. Glidden C. E. Borchardt G. J. Soma T. (2007). Mamu-B^∗^08-positive macaques control simian immunodeficiency virus replication. *J. Virol.* 81 8827–8832. 10.1128/jvi.00895-07 17537848 PMC1951344

[B23] Loffredo J. T. Sidney J. Wojewoda C. Dodds E. Reynolds M. R. Napoe G. (2004). Identification of seventeen new simian immunodeficiency virus-derived CD8 + T cell epitopes restricted by the high frequency molecule, Mamu-A^∗^02, and potential escape from CTL recognition. *J. Immunol.* 173 5064–5076. 10.4049/jimmunol.173.8.5064 15470050

[B24] McDermott A. B. Mitchen J. Piaskowski S. De Souza I. Yant L. J. Stephany J. (2004). Repeated low-dose mucosal simian immunodeficiency virus SIVmac239 challenge results in the same viral and immunological kinetics as high-dose challenge: a model for the evaluation of vaccine efficacy in nonhuman non-human primates. *J. Virol.* 78 3140–3144. 10.1128/jvi.78.6.3140-3144.2004 14990733 PMC353751

[B25] McGary C. S. Alvarez X. Harrington S. Cervasi B. Ryan E. S. Iriele R. I. (2017). The loss of CCR6 (+) and CD161 (+) CD4 (+) T-cell homeostasis contributes to disease progression in SIV-infected rhesus macaques. *Mucosal Immunol.* 10 1082–1096. 10.1038/mi.2016.116 28051083 PMC5474141

[B26] Miller C. J. Alexander N. J. Sutjipto S. Lackner A. A. Gettie A. Hendrickx A. G. (1989). Genital mucosal transmission of simian immunodeficiency virus: animal model for heterosexual transmission of human immunodeficiency virus. *J. Virol.* 63 4277–4284. 10.1128/jvi.63.10.4277-4284.1989 2778875 PMC251042

[B27] Miller C. J. Li Q. Abel K. Kim E. Y. Ma Z. M. Wietgrefe S. (2005). Propagation and dissemination of infection after vaginal transmission of simian immunodeficiency virus. *J. Virol.* 79 9217–9227. 10.1128/jvi.79.14.9217-9227.2005 15994816 PMC1168785

[B28] Nishimura Y. Gautam R. Chun T. W. Sadjadpour R. Foulds K. E. Shingai M. (2017). Early antibody therapy can induce long-lasting immunity to SHIV. *Nature* 543 559–563. 10.1038/nature21435 28289286 PMC5458531

[B29] Pal R. Venzon D. Letvin N. L. Santra S. Montefiori D. C. Miller N. R. (2002). ALVAC-SIV-gag-pol-env-based vaccination and macaque major histocompatibility complex class I (A^∗^01) delay simian immunodeficiency virus SIVmac-induced immunodeficiency. *J. Virol.* 76 292–302. 10.1128/jvi.76.1.292-302.2002 11739694 PMC135699

[B30] Sahu G. K. Chen J. J. Huang J. C. Ramsey K. M. Cloyd M. W. (2001). Transient or occult HIV-1 infection in high-risk adults. *AIDS* 15 1175–1177. 10.1097/00002030-200106150-00013 11416720

[B31] Sahu G. K. McNearney T. Evans A. Turner A. Weaver S. Huang J. C. (2005). Transient or occult HIV infections may occur more frequently than progressive infections: changing the paradigm about HIV persistence. *Arch. Virol. Suppl.* 19 131–145. 10.1007/3-211-29981-5_11 16355871

[B32] Shacklett B. L. Ferre A. L. (2011). Mucosal immunity in HIV controllers: the right place at the right time. *Curr. Opin. HIV AIDS* 6 202–207. 10.1097/COH.0b013e3283453e2b 21399497 PMC3245199

[B33] Silver Z. A. Watkins D. I. (2017). The role of MHC class I gene products in SIV infection of macaques. *Immunogenetics* 69 511–519. 10.1007/s00251-017-0997-3 28695289 PMC5537376

[B34] Sui Y. Lewis G. K. Wang Y. Berckmueller K. Frey B. Dzutsev A. (2019). Mucosal vaccine efficacy against intrarectal SHIV is independent of anti-Env antibody response. *J. Clin. Invest.* 129 1314–1328. 10.1172/JCI122110 30776026 PMC6391089

[B35] Sutton M. S. Burns C. M. Weiler A. M. Balgeman A. J. Braasch A. Lehrer-Brey G. (2016). Vaccination with live attenuated simian immunodeficiency virus (SIV) protects from mucosal, but not necessarily intravenous, challenge with a minimally heterologous SIV. *J. Virol.* 90 5541–5548. 10.1128/JVI.00192-16 26962218 PMC4886799

[B36] Tasca S. Tsai L. Trunova N. Gettie A. Saifuddin M. Bohm R. (2007). Induction of potent local cellular immunity with low dose X4 SHIV(SF33A) vaginal exposure. *Virology* 367 196–211. 10.1016/j.virol.2007.05.021 17574643 PMC2756750

[B37] Tebit D. M. Ndembi N. Weinberg A. Quinones-Mateu M. E. (2012). Mucosal transmission of human immunodeficiency virus. *Curr. HIV Res.* 10 3–8. 10.2174/157016212799304689 22264040 PMC3744389

[B38] Theze J. Chakrabarti L. A. Vingert B. Porichis F. Kaufmann D. E. (2011). HIV controllers: a multifactorial phenotype of spontaneous viral suppression. *Clin. Immunol.* 141 15–30. 10.1016/j.clim.2011.07.007 21865089 PMC3183253

[B39] Xiao P. Patterson L. J. Kuate S. Brocca-Cofano E. Thomas M. A. Venzon D. (2012). Replicating adenovirus-simian immunodeficiency virus (SIV) recombinant priming and envelope protein boosting elicits localized, mucosal IgA immunity in rhesus macaques correlated with delayed acquisition following a repeated low-dose rectal SIV (mac251) challenge. *J. Virol.* 86 4644–4657. 10.1128/JVI.06812-11 22345466 PMC3318604

[B40] Xu H. Wang X. Veazey R. S. (2013). Mucosal immunology of HIV infection. *Immunol. Rev.* 254 10–33. 10.1111/imr.12072 23772612 PMC3693769

[B41] Xue J. Cong Z. Xiong J. Wang W. Jiang H. Chen T. (2013). Repressive effect of primary virus replication on superinfection correlated with gut-derived central memory CD4 (+) T cells in SHIV-infected Chinese rhesus macaques. *PLoS One* 8:e72295. 10.1371/journal.pone.0072295 24023734 PMC3759369

[B42] Yant L. J. Friedrich T. C. Johnson R. C. May G. E. Maness N. J. Enz A. M. (2006). The high-frequency major histocompatibility complex class I allele Mamu-B^∗^17 is associated with control of simian immunodeficiency virus SIVmac239 replication. *J. Virol.* 80 5074–5077. 10.1128/jvi.80.10.5074-5077.2006 16641299 PMC1472056

